# Bioinspired Synthesis of Mesoporous Gold-silica Hybrid Microspheres as Recyclable Colloidal SERS Substrates

**DOI:** 10.1038/s41598-017-15225-8

**Published:** 2017-11-07

**Authors:** Ho Yeon Son, Kyeong Rak Kim, Jun Bae Lee, Trang Huyen Le Kim, Jihui Jang, Su Ji Kim, Moung Seok Yoon, Jin Woong Kim, Yoon Sung Nam

**Affiliations:** 1 Department of Materials Science and Engineering, Korea Advanced Institute of Science and Technology, 291 Daehak-ro, Yuseong-gu, Daejeon, 34141 Republic of Korea; 2 Innovation Lab, Cosmax Research & Innovation Center, 662 Sampyong-dong, Bundang-gu, Seongnam, Gyeonggi-do 13486 Republic of Korea; 3 Department of Bionano Technology, Hanyang University, 55 Hanyangdaehak-ro, Sangnok-gu, Ansan, Gyeonggi-do 15588 Republic of Korea; 4 Department of Chemical and Molecular Engineering, Hanyang University, 55 Hanyangdaehak-ro, Sangnok-gu, Ansan, Gyeonggi-do 15588 Republic of Korea; 5 KAIST Institute for the NanoCentury, Korea Advanced Institute of Science and Technology, 291 Daehak-ro, Yuseong-gu Daejeon, 34141 Republic of Korea

## Abstract

Noble metal nanostructures have been intensively investigated as active substrates for surface-enhanced Raman spectroscopy (SERS) from visible to near-IR wavelengths. However, metal nanoparticle-based SERS analysis in solutions is very challenging due to uncontrollable and irreproducible colloid aggregation. Here we report the templated synthesis of porous gold-silica hybrid microspheres and their application as reusable colloidal SERS substrates. Mesoporous polymer microspheres are synthesized and used as templates for the synthesis of non-aggregated gold nanoparticles, followed by polydopamine-mediated silicification to fabricate mesoporous gold-silica hybrid microspheres. The mesoporous hybrid particles detect crystal violet in the order of 10^–8^ M and provide the structural durability of the immobilized gold nanoparticles, allowing them to be recycled for repeated SERS analyses for analytes in a solution with the similar sensitivity. This work suggests that the mesoporous gold-silica hybrid microspheres are attractive SERS substrates in terms of reusability, sensitivity, and stability.

## Introduction

Raman scattering has received much attention due to its high selectivity and applicability for a wide range of analytes. Although the small cross sections may limit its practical applications, the sensitivity can be efficiently enhanced by the increased electric field at the surface of plasmonic nanostructures^1,2^. The interaction of light with the plasmonic antennas made from metal nanostructures permits the excitation of collective electron oscillation at their surface with the same frequency as the incident light, increasing the intensity by several orders of magnitude^3^. Various metallic nanostructures have been investigated for surface enhanced Raman spectroscopy (SERS)-based sensing applications because their plasmon resonance frequencies range from visible to near-IR regions, which are easily accessible by commercial lasers^4–7^. The electromagnetic enhancement by surface plasmon resonance (SPR) is affected by the composition, size and shape of metal nanostructures, and the dielectric constant of the surrounding medium^8^.

Colloidal metal nanoparticles have been extensively studied as SERS substrates due to the cost effectiveness and versatile surface functionalization of solution-based synthesis. To generate hot spots, the formation of inter-particle junctions through the assembly of metal nanoparticles has been widely examined^9^. However, the particle assembly is often very unpredictable, and the assembled particles are not structurally stable. To solve this problem, the encapsulation of metal nanoparticles within polymer or silica shells has been investigated^10–13^. In this case, analytes can be co-encapsulated within the shell for indirect detection as SERS tags, which improves the sensitivity by confining the location of the analytes in the hotspots. However, this approach is often limited by the difficulty of the formation of controllable and reproducible hotspots in colloidal systems. More recently, porous coatings were introduced to make the surface of metal nanoparticles accessible to analytes while preventing their non-specific aggregation^7^. Mesoporous silica shells have been widely used for this purpose because silica is mechanically stable, chemically and biologically inert, and easily dispersed in aqueous media.

A variety of silica nanostructures, often with high porosity and hierarchical structures, have been developed via biomimetic silicification using organic templates. In particular, the use of porous polymeric structures as sacrificial templates for silicification is very attractive due to their large surface area, low bulk density, and structural uniqueness^14–21^. Porous polymer structures can be prepared using various techniques, including phase separation^18,22–24^, porogen leaching^18,25,26^, gas foaming^27^, electrospinning^20,21,28–31^, and colloidal templating^32^. If metal nanoparticles are incorporated into porous polymer materials with distances of tens of nanometers for plasmonic coupling, silicification can fix the nanostructures while generating ‘negatively replicated’ porous structures that allows the diffusion of analytes to metal nanoparticles. Several methods can be used to synthesize the porous metal-polymer hybrid materials, including the chemical reduction of metal ion salts in a polymer matrix, the *in situ* polymerization in the presence of inorganic nanoparticles, and the dispersion or blending of metal nanoparticles with pre-synthesized polymers^15,23,26,29,30,33–35^.

**Figure 1:**
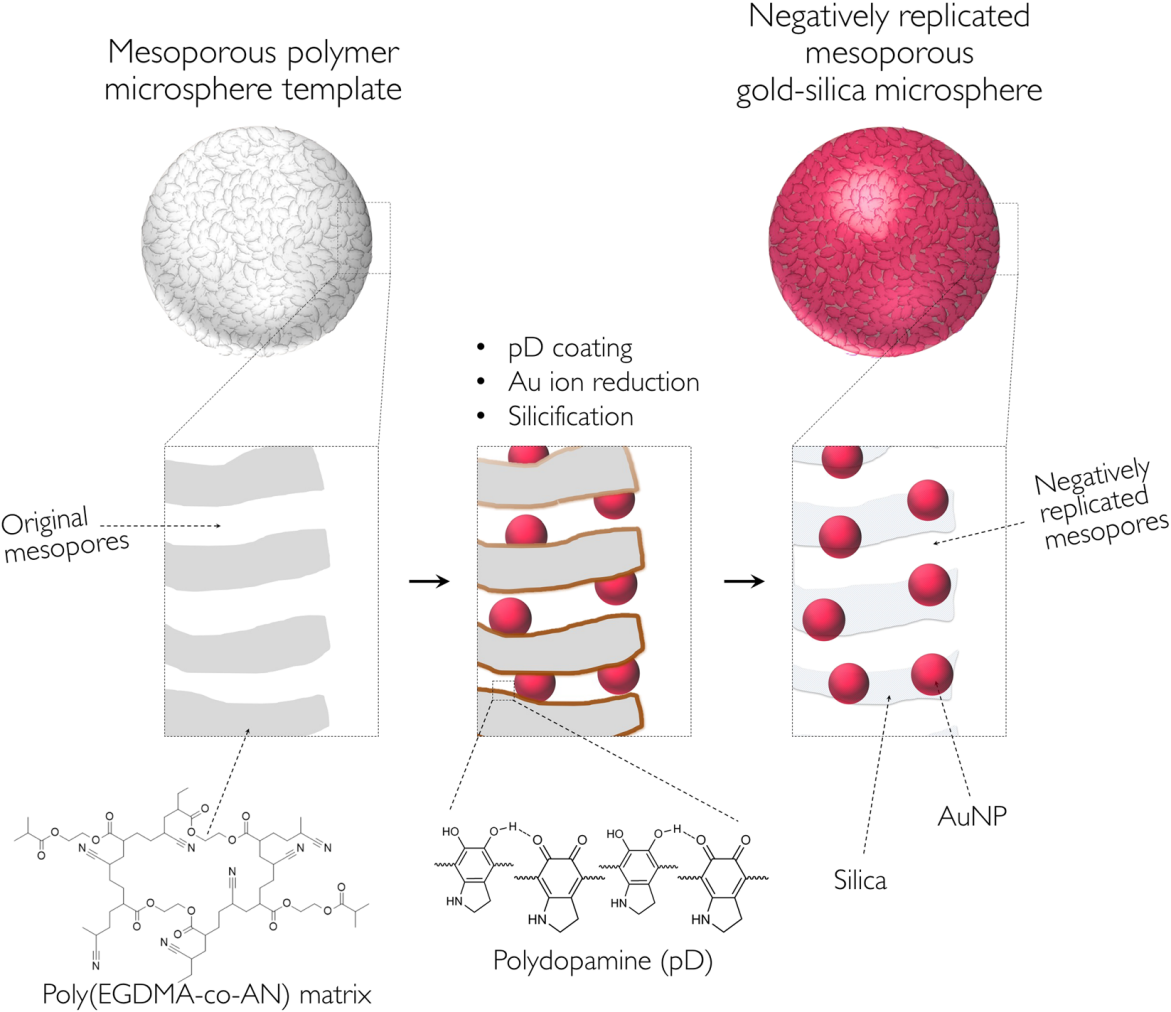
Schematic illustration of the ‘negative replication’ procedures for mesoporous gold-silica hybrid microspheres.

In this study, we introduce a ‘negative replication’ method to fabricate mesoporous gold-silica hybrid microspheres as reusable colloidal SERS substrates using pre-synthesized polymer microsphere templates with mussel-inspired adhesion chemistry, as illustrated in Fig. 1. This synthetic scheme enables the synthesis of gold nanostructures on the surface of nanoscale pores of silica microparticles, which are clearly different from gold nanostructures encapsulated within a silica shell. The mesoporous gold-silica hybrid microspheres prepared by the negative replication method allows the gold surface to be accessible to analytes, making it attractive for SERS applications. The surface functionalization and the following deposition of inorganic materials are based on the oxidative polymerization of catecholamines to polydopamine (pD), which forms a very stable, adhesive protein-like layer^26,29,30,36,37^. The catechol moiety of pD can react with amine and thiol groups by Michael-type addition reactions or Schiff-base formations^38^. The catechol group has a moderate reduction capability with a redox potential of 530 mV vs. reversible hydrogen electrode (RHE) at pH 7^39^. This reduction potential is high enough to reduce noble metal ions into solid metal structures. The pD coatings can also mediate the hydrolysis and condensation of silicate precursors and work as structure-guiding templates for biosilicification.

Highly porous poly(ethylene glycol dimethacrylate-co-acrylonitrile) (poly(EGDMA-co-AN)) microspheres were synthesized by suspension polymerization and used as an architecture template for the *in situ* synthesis of AuNPs and a silica matrix^33,35^. Suspension polymerization was used because it is a very simple method to prepare polymer microspheres having a wide range of porosities and morphologies with different monomeric compositions^15,33–35^. Nitrile groups were incorporated to facilitate the adsorption of chloroaurate ions (AuCl_4_^-^) within the internal surfaces of porous polymer microspheres as demonstrated in our previous work^35,40,41^. The pD coatings of the microspheres enabled the reduction of the deposited gold ions and the following biosilicification, resulting in the formation of mesoporous gold-silica hybrid microspheres. We examined different procedures and compositions to control the structural properties of the gold-silica hybrid microspheres and evaluated their applicability as reusable colloidal SERS substrates using crystal violet (CV) as a model analyte in a solution.

## Results and Discussion

### Synthesis of Mesoporous Poly(EGDMA-co-AN) Microspheres

**Figure 2:**
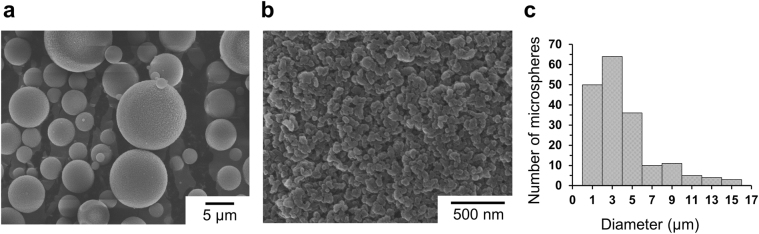
SEM images of the spherical shape (**a**) and porous surface morphology (**b**), and size distribution (**c**) of the synthesized mesoporous poly(EGDMA-co-AN) microspheres.

Mesoporous poly(EGDMA-co-AN) microspheres were synthesized by suspension polymerization using toluene as a porogen according to our previous reports^33,35^. The porous structure was generated by the non-solvent induced phase separation of the synthesized oligomers in the suspended monomer droplets during the polymerization. Scanning electron microscopy (SEM) images of the synthesized polymer microspheres exhibit a spherical shape and porous surface morphology (Fig. 2a,b). The average diameter ± standard derivation of the polymer microspheres was 4.1 ± 3.1 μm (Fig. 2c). The specific surface area of the porous poly(EGDMA-co-AN) microspheres was 136.3 m^2^ g^−1^ as determined by Brunauer–Emmett–Teller (BET) analysis using nitrogen gas^42^. The estimated total pore volume (*V*_*p*_) was 0.454 cm^3^ g^−1^, and the average pore diameter (*D*_*p*_) was 13.3 nm as calculated by the equation, *D*_*p*_ = 4*V*_*p*_/*S*_BET_, where *S* is the BET surface area. The pore volume distribution against pore diameter was obtained by the Barrett, Joyner, and Halenda (BJH) method (Fig. S1)^43^. The result indicates that most of the pore volume of the microspheres was occupied by the mesopores of less than 50 nm in diameter. The high surface area and small pore size of the produced poly(EGDMA-co-AN) microspheres make them very useful templates for the assembly of metal nanoparticles through the deposition of metal ions.

In addition to the porous structures, the surface chemistry of internal pores of the microspheres is also important for the efficient infiltration and adsorption of metal ions. Our previous works demonstrated that the adsorption of metal ions was efficiently facilitated by the nitrile functionalization of the pore surface of polymer microspheres^35^. Accordingly, poly(EGDMA-co-AN) was employed as a matrix polymer in this work by the addition of AN as a co-monomer during the suspension polymerization of poly(EGDMA). The presence of nitrile groups in the synthesized poly(EGDMA-co-AN) microspheres was confirmed using Fourier transform infrared spectroscopy (FT-IR). A characteristic peak at 2240 cm^−1^ was assigned to the C≡N stretching vibration, as represented in Fig. S2 ^35^.

### Polydopamine (pD) Coatings of Mesoporous Microspheres

**Figure 3:**
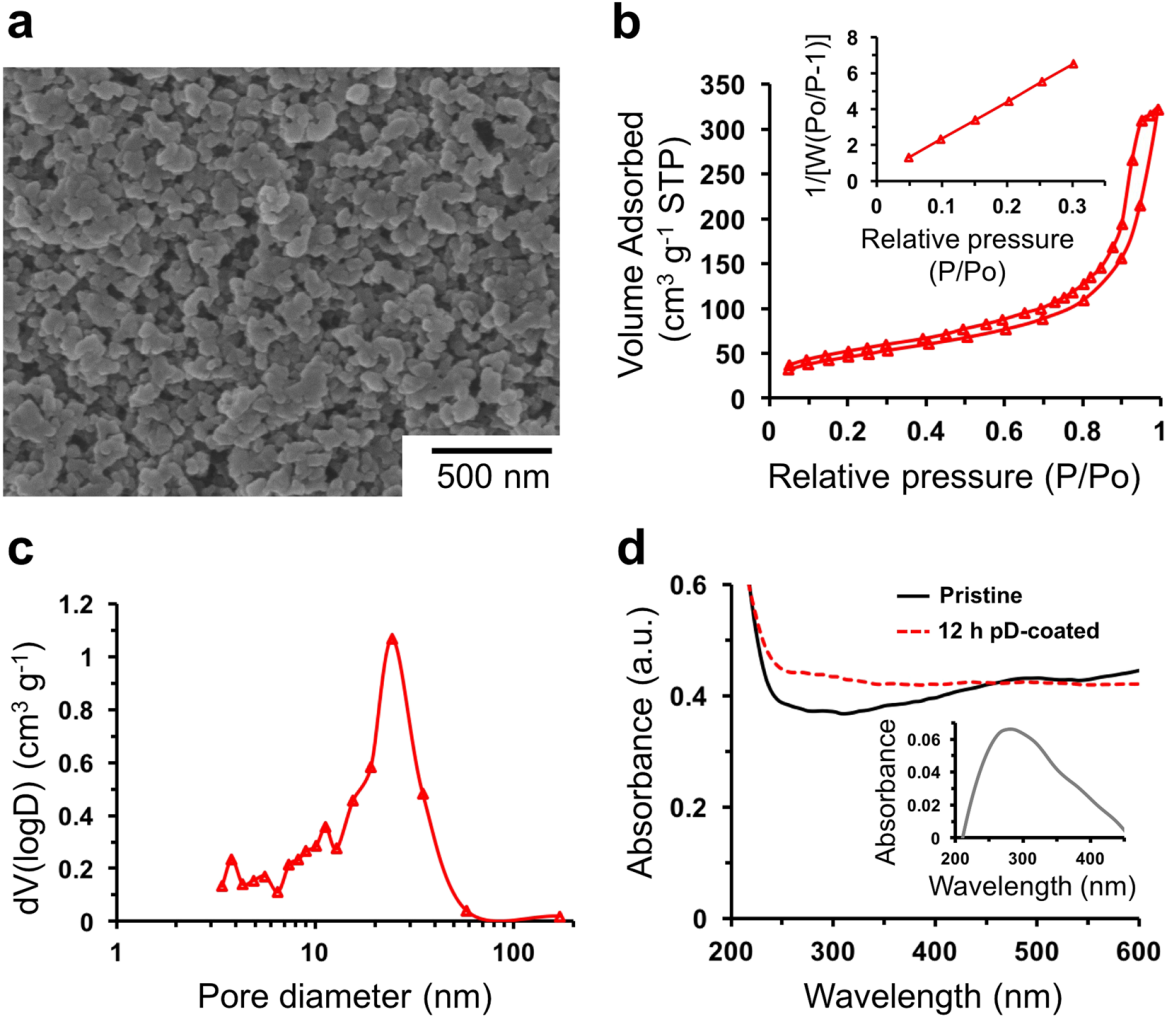
SEM images (**a**), isotherms of N_2_ adsorption (**b**), inset: BET surface area plots), and pore volume distribution (**c**) of the 12 h pD-coated poly(EGDMA-co-AN) microspheres. UV-Vis absorption spectra of the pristine (black) and 12 h pD-coated (red) microspheres dispersed in water at a concentration of 1 mg mL^−1^ (**d**, inset: a spectrum subtracting the spectrum of the pristine from the spectrum of the 12 h pD-coated microspheres).

More rigorous surface modification of the mesoporous microspheres was carried out through pD coatings. The coating procedures are very simple: the microspheres were dispersed in a 70% ethanolic solution of dopamine at an ambient temperature. The oxidative polymerization of dopamine to pD was initiated by adding a small amount of sodium hydroxide. Increasing temperature or pH can result in a very thick deposition of pD, so it is important to keep the mild condition to avoid the blocking of the pores with pD. SEM image of the pD-coated microspheres shows mesoporous surface morphology similar as that of the pristine microspheres (Fig. 3a). However, the specific surface area and the total pore volume of the pD-coated microspheres were 166.5 m^2^ g^−1^ and 0.498 cm^3^ g^−1^, respectively, which are significantly increased compared to the pristine microspheres (Fig. 3b). The results can be attributed to the formation of the pD aggregates that can form the porous structures with granules of the poly(EGDMA-co-AN) on the outer surfaces of the microspheres^26,44,45^. The average pore diameter was decreased to 12.0 nm because the large pores with the diameters larger than 50 nm in the pristine microspheres became smaller due to the pD layers deposited within the pores, as shown in the pore volume distribution obtained by the BJH method (Fig. 3c). The UV-Vis absorption spectra showed the increased absorbance in the pD-coated microspheres around 280 nm due to the catechol groups in the pD layer (Fig. 3d). All of these analyses indicate the successful formation of the pD layer on the poly(EGDMA-co-AN) microspheres via the oxidation polymerization of dopamines.

### *In Situ* Synthesis of AuNPs within Mesoporous Microspheres

**Figure 4:**
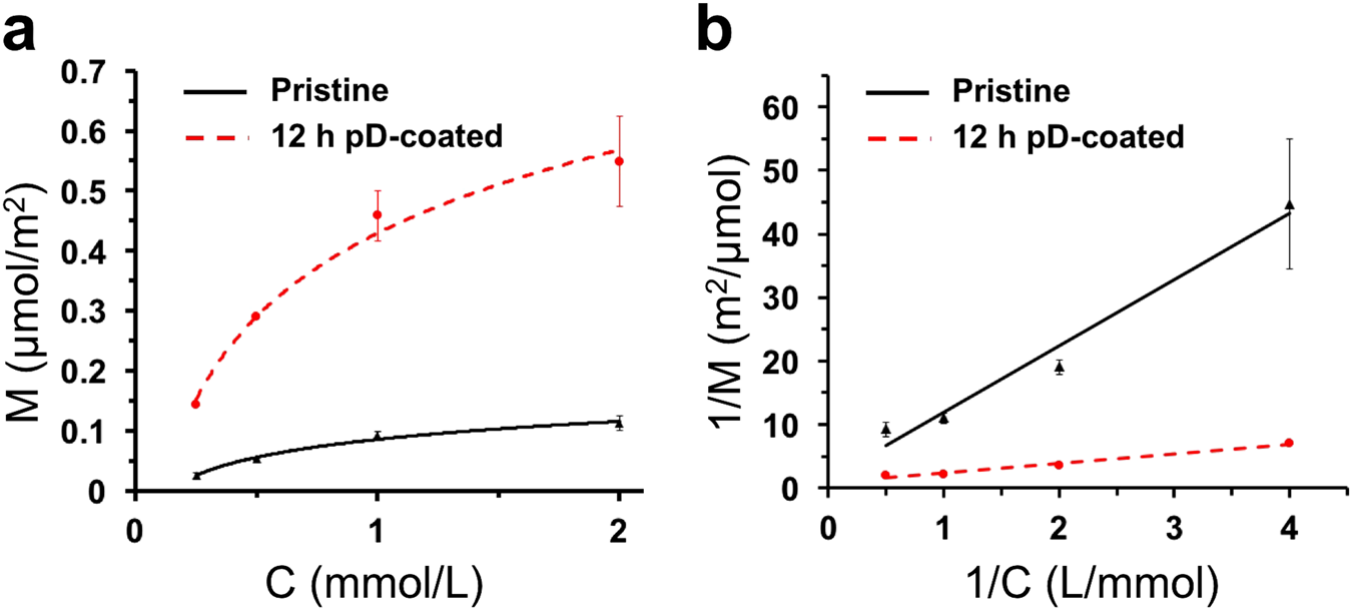
Langmuir isotherm curves (**a**) and Lineweaver-Burk plots (**b**) for adsorption of chloroaurate ions on the pristine (black, triangle) and 12 h pD-coated (red, circle) poly(EGDMA-co-AN) microspheres. Symbols represent the means and standard deviations (n = 3).

The effects of pD coatings on the adsorption of chloroaurate ions in deionized water were examined. The adsorption isotherm of chloroaurate ions on the surface of pristine poly(EGDMA-co-AN) microspheres exhibited Langmuir-type adsorption, as represented in the Lineweaver-Burk regression of the adsorption isotherm (Fig. 4). The calculated binding constant (*K*) was about 1.33 × 10^−4^ L μmol^−1^. The maximum number of chloroaurate ions adsorbed per unit area of the mesoporous substrates (*Г*_*max*_), indicated by the inverse of the *y*-intercept, was 0.72 μmol m^−2^. This value indicates that a single gold ion binds per 2.3 nm^2^ of the poly(EGDMA-co-AN) surface. The total number of the nitrile groups incorporated to the polymer backbone was 3.14 mmol g^−1^, corresponding to 23 μmol m^−2^ if the specific surface area obtained from the BET analysis was used for calculation. The number of the nitrile groups per unit area is larger than *Г*_*max*_, which indicates that a large number of the nitrile groups were not exposed to the pore surface, or the interaction between the nitrile groups and chloroaurate ions was insufficient to induce the stable adsorption of chloroaurate ions on the polymer surface. Despite different molecular interactions, the adsorption isotherm of chloroaurate ions on the pD-coated polymer microspheres also exhibited Langmuir-type adsorption with *K* = 5.57 × 10^−4^ L μmol^−1^ and *Г*_*max*_ = 1.20 μmol m^−2^ (Fig. 4), which corresponded to about 4.2 times and 1.7 times compared to those of pristine microspheres, respectively. These results indicate that the pD layers can efficiently mediate the adsorption of chloroaurate ions on the surface via Au ion-catechol complexation^46^, which result in the increased binding affinity compared to the pristine poly(EGDMA-co-AN) microspheres.

**Figure 5:**
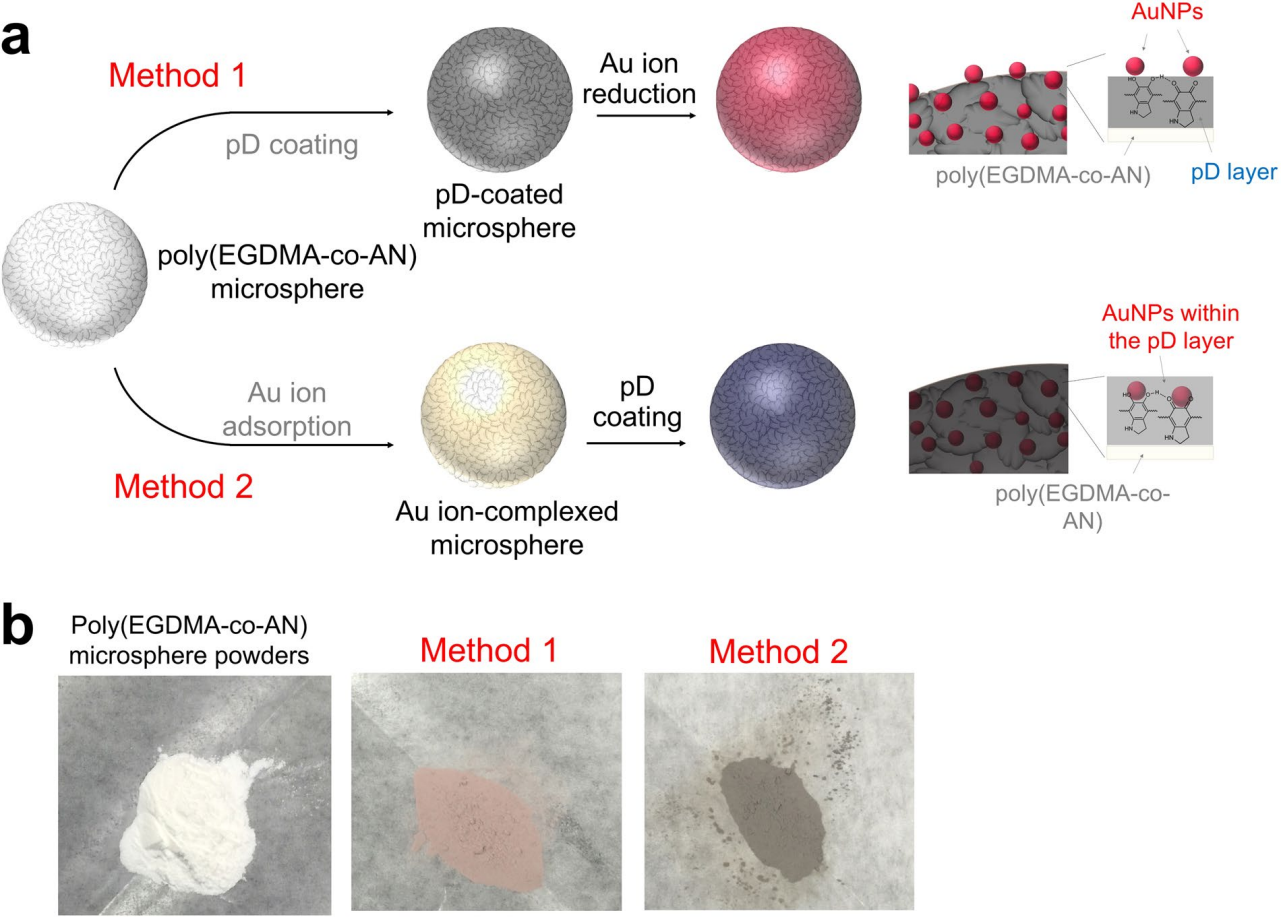
Schematic description of the two different preparation procedures of Au-poly(EGDMA-co-AN) hybrid microspheres. Method 1: pD coating was carried out prior to the deposition of Au ion precursors. Method 2: Au ions were deposited on the internal pores of the microspheres followed by pD coatings (**a**). Photographs of dried powders of pristine poly(EGDMA-co-AN) microspheres and Au-poly(EGDMA-co-AN) hybrid microspheres prepared by method 1 and 2 (**b**).

In addition to the adsorption of chloroaurate ions, the catechol group of pD has a reducing power enough to induce their spontaneous reduction into solid AuNPs because catechol has a redox potential of 500–550 mV vs. RHE at neutral pHs^39^. Recent studies reported the use of catechol and its derivatives for the synthesis of noble metal nanostructures in solutions and on the surface of polymer microparticles and nanofibers^26,29,30,47^. In this work, we extended the application of pD chemistry to the *in situ* formation of AuNPs within the polymer microsphere templates using two different preparation methods, as described in Fig. 5a. In method 1, pD coating was carried out for the mesoporous poly(EGDMA-co-AN) microspheres, followed by the spontaneous reduction of chloroauric acid. In method 2, the sequence was reversed. The poly(EGDMA-co-AN) microspheres were immersed in a solution of chloroauric acid and dried in air, followed by pD coatings. The pD coatings could induce the reduction of the adsorbed Au ions into solid AuNPs. It was hypothesized that these two methods result in different distributions of AuNPs within the mesoporous poly(EGDMA-co-AN) microspheres. Because the pD layer generates a reductive microenvironment for the Au ions, AuNPs can be synthesized on the surface of the pD layer in method 1, while method 2 was expected to produce AuNPs within the pD layer because the adsorbed Au ion precursors need to be dissociated from the surface and diffuse to the pD layer for reduction to solid metal nanoparticles. The color of the dried microspheres indicates the different distributions of AuNPs and pD in the microspheres, as shown in Fig. 5b.

### Gold-Poly(EGDMA-co-AN) Hybrid Microspheres

**Figure 6:**
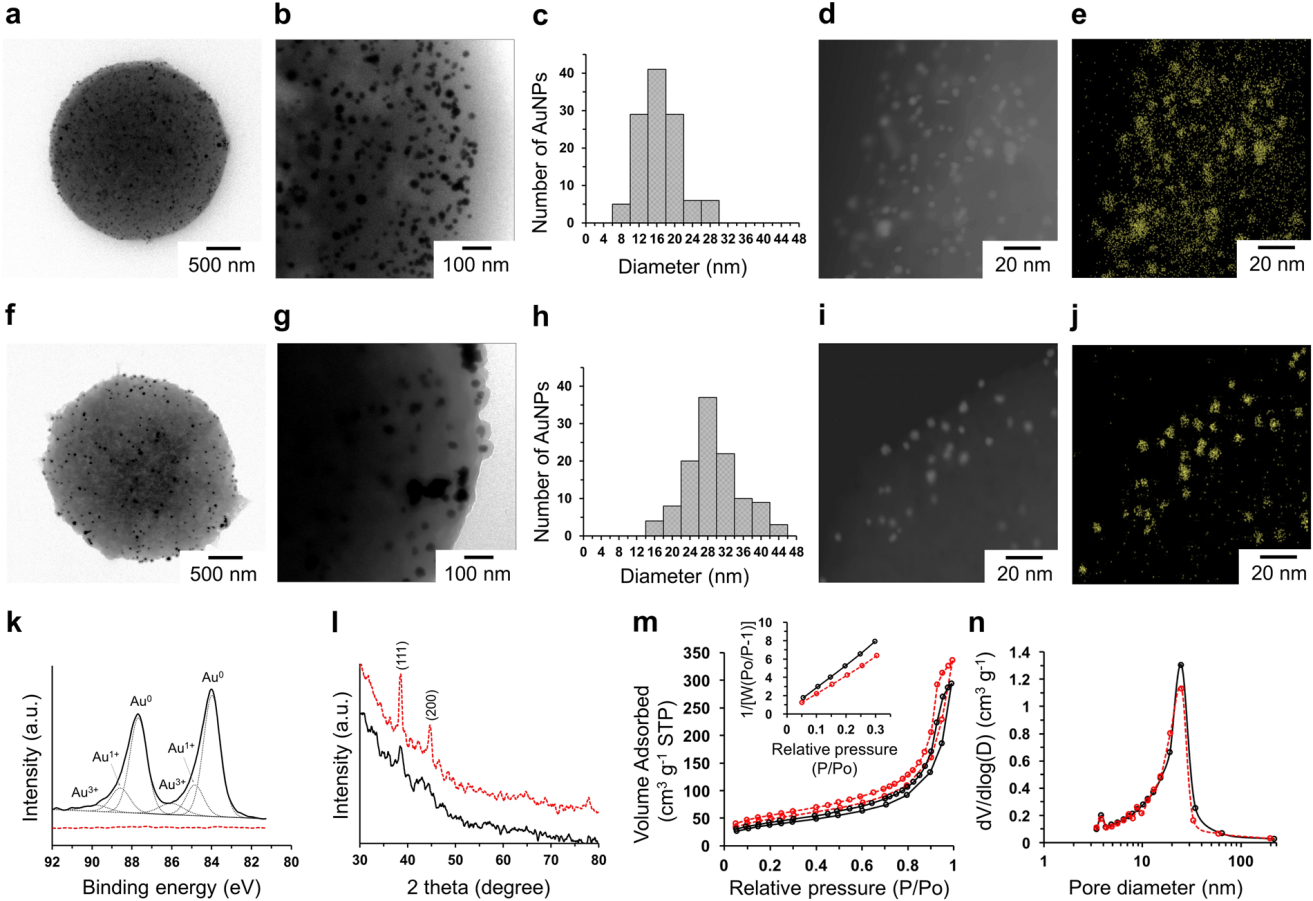
TEM images (**a**, **b**, **f**, and **g**), size distributions (**c** and **h**), STEM images (**d** and **i**), and elemental maps (**e** and **j**) of Au-poly(EGDMA-co-AN) microspheres prepared by method 1 (**a**–**e**) and method 2 (**f**–**j**). XP spectra for Au 4f (**k**), XRD patterns (**l**), isotherms of N_2_ adsorption (**m**, inset: BET surface area plots), and pore volume distribution (**n**) of gold-poly(EGDMA-co-AN) microspheres prepared by method 1 (black) and method 2 (red).

The spatial location and size distribution of AuNPs in the gold-poly(EGDMA-co-AN) hybrid microspheres was examined using TEM, as shown in Fig. 6a–j. The TEM photographs of ultrathin cross-sections of the microspheres, which were prepared by microtoming the samples encapsulated in the frozen section compound, clearly show that AuNPs having a diameter of a few tens of nanometers were synthesized within the porous microspheres. For the negative control prepared by the same procedures using the pristine microspheres instead of the pD-coated microspheres, there was no any AuNPs within the microspheres without a noticeable change in the color of the dried microsphere (Fig. S3). The result indicates that the pD layer acts as a reducing agent, while the nitrile groups on the microspheres do not have enough reducing power for the generation of AuNPs without any addition of reducing agents such as sodium citrate and NaBH_4_^35^. In both cases, method 1 and 2, the AuNPs were homogeneously distributed within the microspheres regardless of the preparation procedures. This result was unexpected because the poly(EGDMA-co-AN) microspheres were coated by the pD layer, which can hinder the diffusion of Au ion precursors and eventually reduce their oxidation number. Moreover, the diffusion through the pores should be much slower than its diffusion in a bulk phase^48^. In our work, the presence of AuNPs in the central part of the microspheres indicates that the reduction kinetics of Au ion precursors was much slower than their hindered diffusion into the microspheres. To clearly compare the size and morphology of the AuNPs, scanning transmission electron microscopy (STEM) analysis with elemental mapping was carried out, as shown in Fig. 6d, e, i, and j. The bright dots in the high magnification STEM images were the AuNPs as indicated by the elemental maps. The particle size distributions of the AuNPs were determined by directly measuring the diameters of at least 100 AuNPs in the STEM images. The average diameters ± standard deviations of the AuNPs were 16.9 ± 4.4 nm and 29.6 ± 6.4 nm for method 1 and 2, respectively. The larger size of the AuNPs by method 2 might be caused by the longer reaction time that leads to the broad size distribution of AuNPs.

The surface chemistry of the gold-poly(EGDMA-co-AN) microspheres was examined using X-ray photoelectron spectroscopy (XPS). Au^0^ 4f peaks were observed only from the hybrid microspheres prepared by method 1 at 87.7 eV and 84.0 eV with peaks for Au^1+^ and Au^3+^ due to Au ion-catechol complexes^46^, while no apparent peaks were found from the sample by method 2 (Fig. 6k). The results indicate that, when the gold-poly(EGDMA-co-AN) hybrid microspheres were synthesized using method 1, the Au ion precursors were reduced on the surface of the hybrid microspheres as well as within the internal pores of the microspheres. However, in the sample prepared by method 2, no significant amount of the Au ion precursors remained on the surface of the microspheres, which were coated by a relatively thick pD layer, as shown in Fig. 6g. The XPS analysis is generally limited to a few nanometer range of the surface of materials due to the relatively short mean free path of the photoelectrons (3–10 nm). In contrast to the XPS results, characteristic X-Ray diffraction (XRD) peaks for AuNPs were clearly observed at 39 ° and 42 ° for (111) and (200) planes from the sample prepared by method 2, while only very small XRD peaks were found from the sample by method 1 (Fig. 6l). The different intensity of the XRD patterns depends on the different particle size of AuNPs. The full width at half maximum (FWHM) of XRD peaks is affected by the particle size according to the Scherrer equation: *D* = *Kλ* / *βcosθ*, where *D* is the particle size, *K* is a constant equal to 0.94, *λ* is Cu *Kα* radiation wavelength (0.154 nm), *β* is the full width at half maximum (FWHM) of diffraction peak, and *θ* is the diffraction angle. The calculated sizes of AuNPs prepared by method 1 and method 2 are 9.7 nm and 23.8, respectively, determined from (111) plane using the Scherrer equation, which are comparable to the average diameters of the AuNPs determined from the STEM images. We also determined whether or not the *in situ* synthesis of AuNPs within the porous microspheres affected the pore characteristics. Their specific surface area and pore characteristics were measured from the BET isotherm of nitrogen adsorption and desorption. The adsorption-desorption isotherms for the gold-deposited microspheres still exhibited a typical type IV mesoporous sorption behavior (Fig. 6m). The specific surface areas of the gold-poly(EGDMA-co-AN) microspheres were 172.4 m^2^ g^−1^ and 152.4 m^2^ g^−1^ for method 1 and 2, respectively. The total pore volume and mean pore diameter were 0.52 cm^3^ g^−1^ and 12.1 nm for method 1, and 0.477 cm^3^ g^−1^ and 12.5 nm for method 2, respectively. Despite the increased density due to the presence of AuNPs, the gold-poly(EGDMA-co-AN) microspheres prepared by method 1 had a slightly higher surface area, 172.4 m^2^ g^−1^, than the pD-coated poly(EGDMA-co-AN) microspheres (166.5 m^2^ g^−1^). The result indicates that the formation of AuNPs generates additional interparticle spaces on the outer surface of the microspheres that increase the specific surface area and total pore volume of the gold-poly(EGDMA-co-AN) microspheres. In addition, the pore volume with large pore diameters (*d* ~ 35 nm) was decreased in the gold-poly(EGDMA-co-AN) hybrid microspheres because AuNPs were generated within the internal pores, while the pore volume with small pore diameters (*d* < 25 nm) was significantly increased due to the formation of interparticle spaces (Figs 3c and 6n). In constrast, the specific surface area of the gold-poly(EGDMA-co-AN) hybrid microspheres prepared by method 2, 152.4 m^2^ g^−1^, was smaller than that of the pD-coated poly(EGDMA-co-AN) microspheres (166.5 m^2^ g^−1^) because AuNPs were formed within the pD layer and could not generate additional open pores and interspace on the outer surface.

### Synthesis of Gold-Silica Hybrid Microspheres

**Figure 7:**
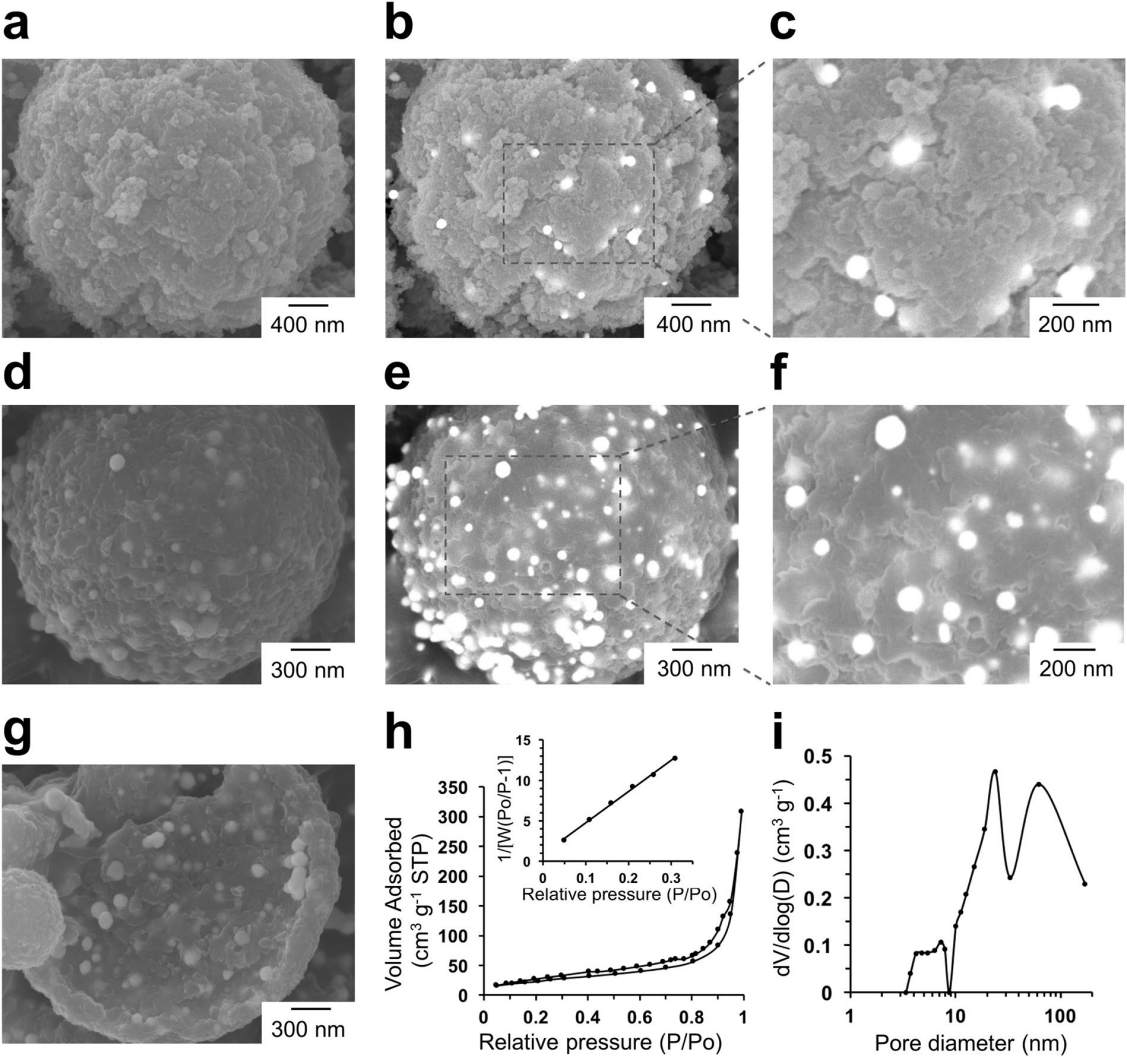
SEM images (left) and BSE-SEM images (middle and right) of gold-silica hybrid microspheres with different silica coating time: 1 h (Au-SiO_2_-mp1) (**a**–**c**) and 30 min (Au-SiO_2_-mp2) (**d**–**f**), and the cross section of Au-SiO_2_-mp2 microspheres (**g**), isotherms of N_2_ adsorption (h, inset: BET surface area plots), and pore volume distribution (**i**) of Au-SiO_2_-mp2.

Although the amounts of chloroaurate ions adsorbed within the mesoporous polymer microspheres with and without pD coatings were similar, the presence of the pD layer highly affected the subsequent silica coating as described in Scheme 1. Without pD modification, the silica coating of the mesoporous polymer microspheres using a silica precursor, tetraethyl orthosilicate (TEOS), was not successful. The hydrolysis and condensation of TEOS in a solution resulted in the formation of silica nanoparticles in the bulk phase rather than surface coatings due to negligible interactions between TEOS and the polymer surface. In contrast, the deposited pD layer within the microspheres efficiently facilitated the deposition of TEOS and the following silica coatings of the microspheres, as represented in Fig. 7. It has been suggested that the indoline structure of pD strongly interacts with silicate species to form a silica-pD hybrid structure^49^. To examine the effects of amount of silica on the morphology and SERS performance, we used three different silicification times (15 min, 30 min, and 1 h), which produced gold-silica hybrid structures with different ratios of silica-to-AuNPs. SEM analyses with backscattered electron (BSE) images exhibit that the silicification time greatly affected the final structure of the gold-silica hybrid microspheres. When it was only 15 min, the negative replication process destroyed all of the microsphere structure into smaller fragments after calcination presumably because the deposited silica was not well interconnected and mechanically fragile. Silicification for 1 h (denoted ‘Au-SiO_2_-mp1’) produced a very thick silica layer on the microspheres as shown in Fig. 7a–c. BSE images (Fig. 7b,c) indicate that most of the AuNPs were embedded inside the external silica layer. In contrast, silicification for 30 min (denoted ‘Au-SiO_2_-mp2′) maintained the integrity of microsphere structures, as represented in Fig. 7d–f, and exhibit a much larger number of AuNPs in the external region of the microsphere. Some blurred AuNPs in the BSE images are the ones embedded in the silica layer. Note that the theoretical depth that can be imaged through BSE is on the order of microns^50^. The cross-sectional BSE image (Fig. 7g) also shows that the AuNPs are broadly distributed within the microspheres. Note that the prepared hybrid structures did not show any structural changes for at least one year when they were stored under ambient conditions. The UV-Vis absorption spectrum of the Au-SiO_2_-mp2 exhibited the absorption peak around 568 nm due to the surface plasmon resonance (SPR) of AuNPs, which was slightly red-shifted compared to the absorption peak of the Au-poly(EGDMA-co-AN) microspheres (546 nm) because of growing the AuNPs as Au ions adsorbed on the pD layer were reduced during the calcination (Fig. S4). The amounts of AuNPs within the hybrid structures were 662 g kg^−1^ and 792 g kg^−1^ for the Au-SiO_2_-mp1 and Au-SiO_2_-mp2, respectevly, as analyzed using inductively coupled plasma mass spectrometry (ICP-MS). The weight ratios of silica-to-AuNP was 0.51:1 and 0.26:1 for the Au-SiO_2_-mp1 and Au-SiO_2_-mp2, respectevly, which clearly shows that the amount of silica was increased with increasing the silification time. The specific surface area and mean pore diameter of the Au-SiO_2_-mp2 was 88.4 m^2^ g^−1^ and 21.6 nm, respectively, determined by BET analysis and BJH method (Fig. 7h and i). The high surface area and porosity suggest that the negatively replicated mesoporous gold-silica microspheres are promising as SERS substrates for analytes in a solution because all of the AuNPs regardless of their location on and within the microsphere are accessible to analytes through hindered diffusion through negatively replicated mesopores.

### SERS Analysis of Gold-Silica Hybrid Microspheres

**Figure 8:**
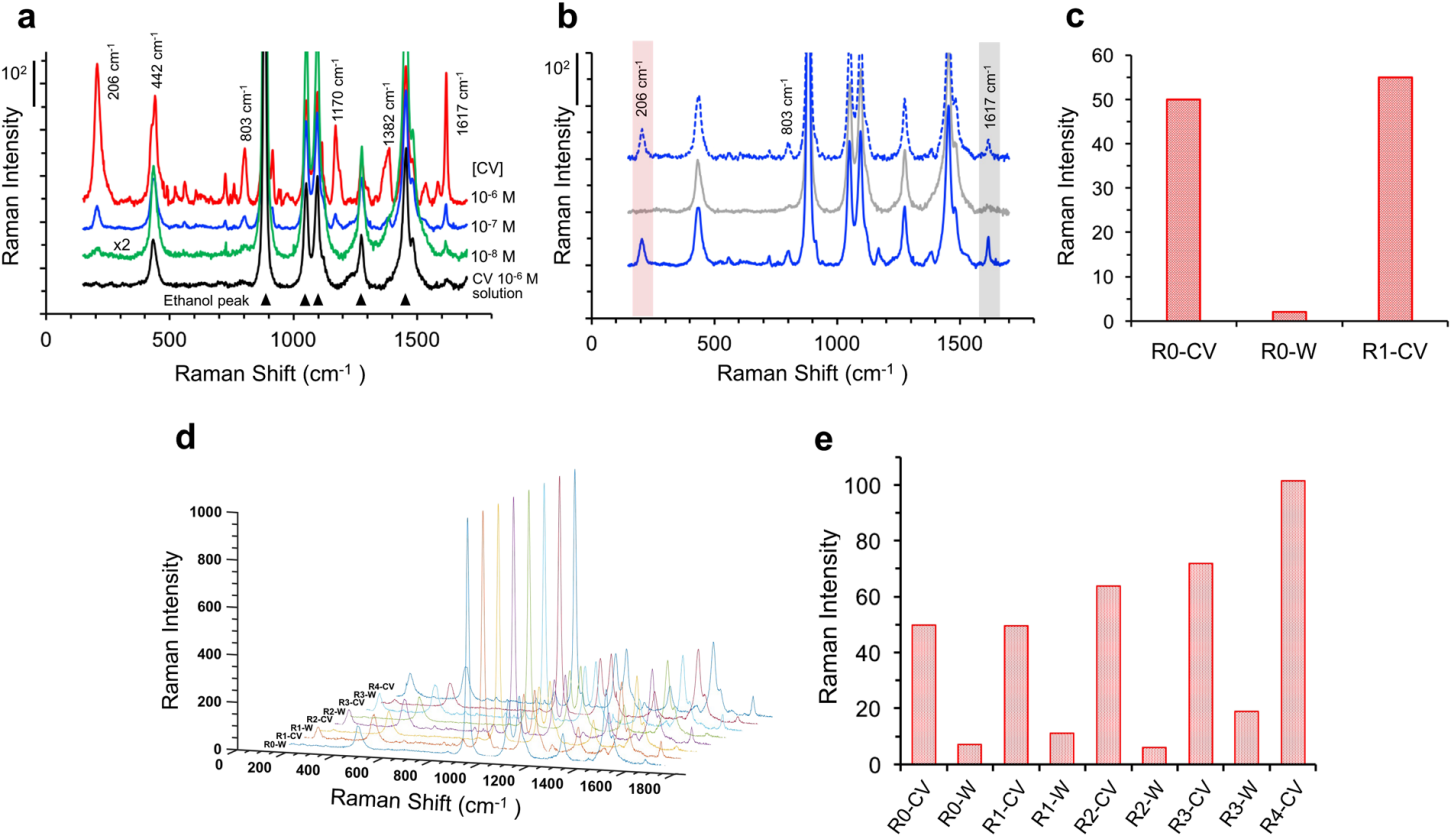
SERS spectra of 10^−n^ M CV (n = 6 (red), 7 (blue), and 8 (green)) with Au-SiO_2_-mp2 ([Au°] = 0.5 mM) and 10^−6^ M CV without Au-SiO_2_-mp2 (black) (**a**). SERS spectra of 10^−7^ M CV with fresh (blue solid line, denoted ‘R0-CV’), regenerated (gray solid line, denoted ‘R0-W’), and reused (blue dashed line, denoted ‘R1-CV’) Au-SiO_2_-mp2 (**b**) and each SERS intensities at 206 cm^−1^ (**c**). Characterization of reusability of Au-SiO_2_-mp2 with four cycles: SERS spectra (**d**) and each SERS intensities at 206 cm^−1^ (**e**) of 10^−7^ M CV with reusable Au-SiO_2_-mp2 in CV solution (R‘n’-CV) and regenerated Au-SiO_2_-mp2 in ethanol (R‘n’-W), where n is the recycled number. Among characteristic peaks for CV, 1617 cm^−1^ is assigned to the vibrational mode of benzene.

Next, we evaluated the SERS performance of colloidal gold-silica microspheres dispersed in ethanol as colloidal SERS substrates and CV as a model analyte. CV carries no functional group that can chemically bind to the substrate. Citrate-stabilized AuNPs with an average dimater of 11.6 nm were used as a negative control. The gold concentration of all of the samples was fixed to 0.5 mM, as measured using ICP-MS. SERS spectra exhibited characteristic peaks of CV: 206 cm^−1^, 442 cm^−1^, 803 cm^−1^, 1170 cm^−1^, 1382 cm^−1^, and 1617 cm^−1^ (Fig. 8). The Raman scattering peaks for ethanol are also shown in the figures, and the peak at 884 cm^−1^ was used for the normalization of the intensities of different SERS spectra. The colloidal Au-SiO_2_-mp2 detected CV in the order of 10^–8^ M (Fig. 8a). We calculated the enhancement factor (*EF*) for the Au-SiO_2_-mp2 using the SERS intensity (*I*_*SERS*_) at 1617 cm^−1^ in the SERS spectrum of 10^–6^ M CV using following equation: *EF* = (*I*_*SERS*_ *·N*_*Raman*_)/(*I*_*Raman*_ *·N*_*SERS*_), where *N*_*SERS*_ and *N*_*Raman*_ are the number of analyte molecules contributing to the SERS signal and the Raman signal, respectively, and *I*_*Raman*_ is the bulk Raman intensity in the Raman spectrum. To estimate the number of CV molecules contributing to the signal, the active surface area of AuNPs in the Au-SiO_2_-mp2 was calculated using the adsorption of 1-dodecanethiol (DDT)^51^. The specific adsorption amount of DDT was 1.27 × 10^−3^ mg per mg of Au-SiO_2_-mp2, measured using high-performance liquid chromatography (HPLC). The active surface area of the AuNPs was calculated using an equation: *a*_*s*_ = *N*_*A*_ *·n*_*s*_/(*θ·ρ*), where *N*_*A*_ is the Avogadro’s number, *n*_*s*_ is the mole of adsorbed DDT, *θ* is the surface coverage of n-alkanethiols on gold, and *ρ* is the surface atom concentration of gold. The *θ* and *ρ* have been known as 0.33 and 11.5 nm^−2^, respectively^51^. The active surface area of AuNPs in the Au-SiO_2_-mp2 was calculated to 9.95 × 10^14^ nm^2^ per 1 mg of Au-SiO_2_-mp. The number of CV molecules adsorbed on the exposed AuNPs in 0.125 mg mL^−1^ of Au-SiO_2_-mp2 used in our experiments was 1.08 × 10^14^, determined using the molecular area of triphenylmethane dye^52^, 1.15 nm^2^. In the case of citrate-stabilized AuNPs, all of the CV molecules can contribute to the signal on the AuNPs ([Au^0^] = 0.5 mM) due to their large surface area, 2.64 × 10^15^ nm^2^, which was calculated using the average diameter (11.6 nm) of the spherical AuNPs. The EF for the citrate-stabilized AuNPs was only 50.5, which is comparable with the results in the previous literature^53^. The *EF* for the Au-SiO_2_-mp2 was 380, which is higher than that for the AuNPs. Interestingly, the SERS intensities and EF values were significantly increased with the increased concentration of Au-SiO_2_-mp2 (Fig. S5). The enhanced EF can be attributed to the mesoporous structures of the Au-SiO_2_-mp2, which can confine the CV solution within the internal pores of the mesoporous structures. The EF for the Au-SiO_2_-mp2 was increased up to 3.16 × 10^3^ at the gold concentration of 2 mM. Furthermore, the mesoporous silica matrix can provide the structural durability of the immobilized gold nanoparticles and allows them to be recycled for repeated SERS analyses in a solution. Figure 8b shows the reusability of the colloidal Au-SiO_2_-mp2 for the CV detection at a CV concentration of 10^−7^ M. The colloidal SERS substrates dispersed in a CV solution were collected by centrifugation and washed using ethanol containing NaOH for 2 h for the desorption of residual CV molecules from the SERS substrate^54^. After the NaOH treatment, the SERS intensities of CV were significantly diminished by about 96% and 80% at 206 cm^−1^ and 1617 cm^−1^, respectively, which indicates that NaOH effectively removed the CV molecules from the surface of the SERS substrate (Fig. 8b–c and Fig. S6a). In addition, the Au-SiO_2_-mp2 maintained show their integrity of microsphere structures without any significant structural changes (Fig. S7). The regenerated Au-SiO_2_-mp2 were reused for the CV detection using 10^−7^ M CV. The SERS signals with the similar sensitivity appeared at 206 cm^−1^ and 1617 cm^−1^. The results clearly show that the colloidal gold-silica microspheres can repeatedly be used for the detection of target molecules with good reproducibility. We further investigated the reusability of the colloid SERS substrates using the short treatment time of NaOH for 10 min (Fig. 8d–e and Fig. S6b). Although the CV molecules cannot be completely removed during the short treatment time, the SERS spectra clearly exhibit the superior reproducibility and recyclability of the colloidal gold-silica microspheres with four cycles. In addition, the gold-silica hybrid microspheres exhibited long-term stability as a powder or dispersed in ethanol before and after SERS analysis.

## Conclusion

We suggested mesoporous gold-silica hybrid microspheres as reuable colloidal SERS substrates in an aqueous solution under ambient conditions. Mesoporous poly(EGDMA-co-AN) microspheres were prepared by suspension polymerization and utilized as templates for the synthesis and deposition of AuNPs and silica matrices. The pD coating of the porous microspheres was carried out via the oxidative polymerization of dopamine at an alkaline pH. The redox-active pD layer greatly improved the adsorption of chloroaurate ions via Au ion-catechol complexation and mediated the spontaneous reduction of Au ion precursors into AuNPs within the mesoporous poly(EGDMA-co-AN) microspheres. In addition, the pD-mediated silicification produced mesoporous gold-silica hybrid microspheres through ‘negative replication’, which retained the mesoporous structures with the high surface area and porosity after the removal of the mesoporous polymer templates by calcination. The SERS performance of the gold-silica hybrid microspheres was investigated using the various concentrations of CV as a model analyte. The SERS signals were detected as low as 10^−8^ M CV with EF values up to 3.16 × 10^3^. The gold-silica hybrid microspheres used for the SERS analyses were regenerated by NaOH treatment and reused for the detection of CV. The SERS signals were repeatedly detected with the similar sensitivity for four cycles. Our bio-inspired approach provides the plasmonic metal-silica hybrid materials that can be utilized as recyclable colloidal SERS substrates for the detection of target molecules with good reproducibility. In addition, since the suspension polymerization using toluene as a porogen allows the large-scale synthesis of the mesoporous poly(EGDMA-co-AN) microspheres, we expect that the mass production of the mesoporous metal-silica hybrid microspheres is highly feasible through a cost-efficient solution-based process.

## Experimental Section

### Materials

Ethylene glycol dimethacrylate (EGDMA), acrylonitrile, dopamine hydrochloride, gold(III) chloride trihydrate (HAuCl_4_^.^3H_2_O, 99.9%), TEOS ( ≥ 99.0%), and CV were purchased from Sigma-Aldrich (St. Louis, MO, USA). Polyvinyl alcohol (PVA, Mw = 8.8–9.2 × 10^4^ g mol^−1^, 87–89% degree of saponification) was purchased from Kuraray Co. (Kurashiki, Okayama, Japan). 2,2′-Azobis(2,4-dimethyl valeronitrile) (ADVN, Wako Pure Chemicals Industries, Ltd, Osaka, Japan) was recrystallized from methanol before use. Sodium hydroxide and ammonia hydrate (28 wt%) were obtained from Junsei Chemical (Tokyo, Japan). Ethanol (94.5%) was purchased from Daejung Chemicals (Siheung, Republic of Korea). Milli-Q water was used as deionized water in all experiments.

### Synthesis of Mesoporous Poly(EGDMA-co-AN) Microspheres

Mesoporous poly(EGDMA-co-AN) microspheres were synthesized by suspension polymerization as described previously^33,35^. Briefly, a mixture of EGDMA, acrylonitrile, ADVN (1 wt% against total monomer), and toluene (40 wt%) was emulsified in a 1 wt% PVA solution using a homogenizer. To induce the formation of internal pores within the microspheres, 40 wt% of toluene was added. The prepared suspension was transferred to a double-walled glass reactor equipped with a stirrer, a reflux condenser, thermocouples, and a nitrogen gas inlet system. The polymerization in the aqueous phase was suppressed using a small amount of sodium nitrite (0.01 wt%). The polymerization was carried out at 60 °C for 10 h at 250 rpm. The synthesized microspheres were collected by filtration, repeatedly washed using methanol, and dried in vacuum.

### *In situ* Synthesis of AuNPs within Mesoporous Microspheres

In method 1, the synthesized poly(EGDMA-co-AN) microspheres (100 mg) were dispersed in 10 mL of ethanol using bath-type sonication for 10 min for infiltration of the solution into the internal pores of the microspheres, and then magnetically stirred for 50 min at room temperature to increase the wettability of the microspheres. After the excess ethanol was removed by centrifugation at 900 × g for 5 min, the microspheres were immersed in 10 mL of a dopamine solution (2 mg mL^−1^) dissolved in a 7:3 (v/v) mixture of ethanol and Milli-Q water. Sodium hydroxide solution (0.1 M) was added into the mixture to be a concentration of 2 × 10^−3^ M for the initiation of the oxidative pD coating. The mixture was dispersed using bath-type sonication for 10 min, and then stirred magnetically in an oil bath at 37 °C for 12 h. The pD-coated microspheres were collected by centrifugation at 900 × g for 5 min and rinsed with about 35 mL of Milli-Q water. As for the synthesis of AuNPs, the pD-coated microspheres (100 mg) were immersed in 10 mL of 0.5 mM HAuCl_4_ solution dissolved in a 7:3 (v/v) mixture of ethanol and Milli-Q water using bath-type sonication (SK7200BT, Shanghai Yanhe Instrument Equipment Co., Ltd., Shanghai, China) for 10 min, followed by magnetic stirring in an oil bath at 80 °C for 10 min. In method 2, the synthesized poly(EGDMA-co-AN) microspheres (100 mg) were pretreated with ethanol for wetting using the same procedure described above. After the excess ethanol was removed, the microspheres were immersed in 1 mL of 5 mM HAuCl_4_ aqueous solution dissolved in a 7:3 (v/v) mixture of ethanol and Milli-Q water using sonication for 10 min, and the mixture was dried in air at room temperature for 24 h. The microspheres treated with a gold precursor were immersed in 10 mL of a dopamine solution (2 mg mL^−1^) dissolved in a 7:3 (v/v) mixture of ethanol and Milli-Q water, and pD coating was conducted with the addition of a NaOH solution at 37 °C for 12 h using the same procedures described above.

### Adsorption of Gold Ion Precursors

The synthesized poly(EGDMA-co-AN) microspheres were treated with ethanol, as described above. Gold ion precursors were prepared by dissolving HAuCl_4_ in a 7:3 (v/v) mixture of ethanol and Milli-Q water at the concentration of 0.25 to 2 mM. The ethanol-treated microspheres and the pD-coated microspheres were added into the HAuCl_4_ solution at a concentration of 10 mg mL^−1^, and then immersed using bath-type sonication for 10 min at room temperature. The microspheres were collected by centrifugation at 900 × g for 5 min, and the supernatants were filtered using Ministar filters with pore diameter of 0.8 µm. The concentration of the remaining chloroaurate ions were measured by the UV-Vis absorbance of the supernatant at 318 nm. The adsorption isotherm was analyzed by the Lineweaver Burk equation:

$${{\Gamma}}^{-{1}}={{{\Gamma}}_{max}}^{-1}+{({\rm{\Gamma}}_{max}KC)}^{-1},$$

where *Г* = mole of the adsorbed chloroaurate ions per unit area, *Г*_*max*_ = mole of the adsorbed chloroaurate ions adsorbed per unit area, *K* = Langmuir equilibrium constant, and *C* = the bulk concentration of chloroaurate ions.

### Synthesis of Gold-silica Hybrid Microspheres

The synthesized polymer microspheres (100 mg) were firstly dispersed in 1 mL of ethanol, and then TEOS (20 μL) was added to the microsphere dispersion with bath-type sonication for 30 min. Ammonia solution (28%, 20 μL) and deionized water (100 μL) were then added, followed by continuous bath-type sonication for 30 min at room temperature. The silica-coated microspheres were collected by centrifugation at 900 × g for 5 min, rinsed with about 35 mL of deionized water three times and dried in vacuum. The dried microspheres were calcinated at 500 °C for 4 h to remove the pD and polymer templates.

### SERS Characterization

The gold-silica hybrid microspheres (Au-SiO_2_-mp2) were dispersed in ethanol at a concentration of 0.25 mg mL^−1^ ([Au°] = 1 mM). The CV was dissolved in ethanol at concentrations of 2 × 10^−n^ M. The prepared Au-SiO_2_-mp2 dispersion (0.5 mL) and CV solution (0.5 mL) were mixed, and sonication for 10 min was performed for the infiltration of CV solution into the mesoporous microspheres. The final concentrations of the mixture were 0.5 mM for gold and 10^−n^ M for CV. For the regeneration of the used Au-SiO_2_-mp2, the Au-SiO_2_-mp2 were collected from the mixture by centrifugation at 3000 × g for 5 min, re-disperse in 1 mL of ethanol. One hundred microliters of 0.05 M NaOH solution was added to the dispersion, washed by sonication for 10 min and magnetic stirring for 2 h, and then collected by centrifugation at 3000 × g for 5 min. The Au-SiO_2_-mp2 were washed with 1 mL ethanol twice, then finally dispersed in 0.5 mL of ethanol. The regenerated Au-SiO_2_-mp2 were mixed with ethanol and 2 × 10^−7^ M of CV solution, respectively, at a volume ratio of 1:1 for the SERS analyses. Raman spectroscopic analyses were carried out using a Dispersive Raman Spectrometer (LabRAM Aramis, Horiba Jobin Yvon, Kyoto, Japan) equipped with 633 nm HeNe laser excitation. The colloidal samples were loaded into microhaematocrit capillary tubes (Marienfeld Laboratory Glassware, 1.1–1.2 mm Ø int., 1.5–1.6 mm Ø ext.), and the laser beam was focused on the sample solution in the capillary tube by a 50× microscope objective lens. Each Raman spectrum was determined from the accumulated value of ten measurements with an exposure time of 5 sec with a laser power of 10 mW.

### Characterization

The morphology of the microspheres was examined using SEM (Hitachi S-4800, Hitachi, Ltd., Tokyo, Japan) at an acceleration voltage of 10 kV after about 3 nm-thick platinum coatings and TEM (Tecnai TF30 ST, FEI, Hillsboro, OR, USA) at an acceleration voltage of 300 kV. The microspheres were encapsulated using a frozen section compound (Leica FSC 22, Leica Microsystems, Wetzlar, Germany) and rapidly frozen in liquid nitrogen. The slices of microspheres were prepared using a cryostat cryocut microtome (Leica CM1850, Leica Microsystems, Wetzlar, Germany) to observe the cross-sectional images. STEM images were obtained on the TEM operating in STEM mode. The elemental composition was analyzed using XPS (Thermo Scientific Sigma Probe, Thermo Fisher Scientific, Inc., Waltham, MA, USA). The XRD patterns of the AuNPs were obtained using a powder X-ray diffractometer (Rigaku, D/MAX-2500). The chemical functional groups of the microspheres were analyzed using Fourier transform infrared spectroscopy (FT-IR, JASCO, Easton, MD, USA). The amounts of AuNPs within the gold-silica hybrid microspheres were quantitatively analyzed using inductively coupled plasma mass spectrometry (ICP-MS, Agilent ICP-MS 7700 S). The surface areas of the porous microspheres were determined by BET analysis using an AUTOSORB-1 analyzer (Quantachrome Corporation, Boynton Beach, FL, USA). To determine the exposed surface area of AuNPs in the Au-SiO_2_-mp2, 26 mg of Au-SiO_2_-mp2 were dispersed in acetonitrile containing DDT at a concentration of 0.05 mg mL^−1^. The solution was magnetically stirred for 20 h, and then the supernatant containing free DDT was separated by centrifugation at 10,000 rpm for 10 min and filtered through a 0.2 μm regenerated cellulose filter. The amount of DDT molecules in the filtrate was analyzed using HPLC (HPLC 1260 series, Agilent Technologies) equipped with a Capcell Pak C18 column (4.6 mm × 250 mm). The mobile phase was acetonitrile. The flow rate was set to 0.5 mL min^−1^ with an injection volume of 100 μL, and eluted peaks were monitored at 227 nm. A calibration curve was obtained using a series of DDT solutions at different concentrations.

## Electronic supplementary material

Supplementary information

## References

[1] Kneipp K (1997). Single molecule detection using surface-enhanced Raman scattering (SERS). Phys. Rev. Lett..

[2] Nie S, Emory SR (1997). Probing single molecules and single nanoparticles by surface enhanced Raman scattering. Science.

[3] Moskovits M (2013). Persistent misconceptions regarding SERS. Phys. Chem. Chem. Phys..

[4] Stamplecoskie KG, Scaiano JC, Tiwari VS, Anis H (2011). Optimal size of silver nanoparticles for surface-enhanced Raman spectroscopy. J. Phys. Chem. C.

[5] Feng H (2009). Simple and rapid synthesis of ultrathin gold nanowires, their self-assembly and application in surface-enhanced Raman scattering. Chem. Commun..

[6] Kedia A, Kumar H, Kumar PS (2015). Tweaking anisotropic gold nanostars: covariant control of a polymer–solvent mixture complex. RSC Adv..

[7] Sanz-Ortiz MN, Sentosun K, Bals S, Liz-Marzán LM (2015). Templated growth of surface enhanced raman scattering-active branched gold nanoparticles within radial mesoporous silica shells. ACS Nano.

[8] Jain PK, Lee KS, El-Sayed IH, El-Sayed MA (2006). Calculated absorption and scattering properties of gold nanoparticles of different size, shape, and composition: applications in biological imaging and biomedicine. J. Phys. Chem. B.

[9] Lim DK (2011). Highly uniform and reproducible surface-enhanced Raman scattering from DNA-tailorable nanoparticles with 1-nm interior gap. Nat. Nanotech..

[10] Chen G (2010). Measuring ensemble-averaged surface-enhanced Raman scattering in the hotspots of colloidal nanoparticle dimers and trimers. J. Am. Chem. Soc..

[11] Li JF (2010). Shell-isolated nanoparticle-enhanced Raman spectroscopy. Nature.

[12] Wang Y, Yan B, Chen L (2012). SERS tags: novel optical nanoprobes for bioanalysis. Chen, Chem. Rev..

[13] Abalde-Cela S (2010). Surface-enhanced Raman scattering biomedical applications of plasmonic colloidal particles. J. R. Soc. Interface.

[14] White RJ, Luque R, Budarin VL, Clark JH, Macquarrie DJ (2009). Supported metal nanoparticles on porous materials. Methods and applications. Chem. Soc. Rev..

[15] Kim JW (2002). Titanium dioxide/poly (methyl methacrylate) composite microspheres prepared by *in situ* suspension polymerization and their ability to protect against UV rays. Colloid Polym. Sci..

[16] Lin VSY, Motesharei K, Dancil KPS, Sailor MJ, Ghadiri MR (1997). A porous silicon-based optical interferometric biosensor. Science.

[17] Zhang D (2015). A highly sensitive and selective hydrogen peroxide biosensor based on gold nanoparticles and three-dimensional porous carbonized chicken eggshell membrane. PLOS ONE.

[18] Nam YS, Park TG (1999). Biodegradable polymeric microcellular foams by modified thermally induced phase separation method. Biomaterials.

[19] Cho KY (2001). Protein release microparticles based on the blend of poly (D, L-lactic-co-glycolic acid) and oligo-ethylene glycol grafted poly (L-lactide). J. Control. Rel..

[20] Lee JA, Nam YS, Rutledge GC, Hammond PT (2010). Enhanced Photocatalytic Activity using Layer‐by‐Layer Electrospun Constructs for Water Remediation. Adv. Funct. Mater..

[21] Li D, Xia Y (2004). Electrospinning of nanofibers: reinventing the wheel?. Adv. Mater..

[22] Nam YS, Park TG (1999). Porous biodegradable polymeric scaffolds prepared by thermally induced phase separation. J. Biomed. Mater. Res..

[23] Lee JE (2004). Polymer/Ag composite microspheres produced by water-in-oil-in-water emulsion polymerization and their application for a preservative. Colloid Polym. Sci..

[24] Lee JS, Kim JW, Kim J, Han SH, Chang IS (2004). Photochemical properties of UV-absorbing chemicals in phase-controlled polymer microspheres. Colloid Polym. Sci..

[25] Kim HK, Chung HJ, Park TG (2006). Biodegradable polymeric microspheres with “open/closed” pores for sustained release of human growth hormone. J Control. Rel..

[26] Son HY (2014). *In situ* functionalization of highly porous polymer microspheres with silver nanoparticles via bio-inspired chemistry. RSC Adv..

[27] Nam YS, Yoon JJ, Park TG (2000). A novel fabrication method of macroporous biodegradable polymer scaffolds using gas foaming salt as a porogen additive. J. Biomed. Mater. Res..

[28] Reneker DH, Yarin AL (2008). Electrospinning jets and polymer nanofibers. Polymer.

[29] Son HY, Ryu JH, Lee H, Nam YS (2013). Silver‐Polydopamine Hybrid Coatings of Electrospun Poly (vinyl alcohol) Nanofibers. Macromol. Mater. Eng..

[30] Son HY, Ryu JH, Lee H, Nam YS (2013). Bioinspired templating synthesis of metal–polymer hybrid nanostructures within 3D electrospun nanofibers. ACS Appl. Mater. Interfaces.

[31] Carlberg B, Ye LL, Liu J (2011). Surface‐Confined Synthesis of Silver Nanoparticle Composite Coating on Electrospun Polyimide Nanofibers. Small.

[32] Johnson SA, Ollivier PJ, Mallouk TE (1999). Ordered mesoporous polymers of tunable pore size from colloidal silica templates. Science.

[33] Kim JW (2004). Synthesis of silver/polymer colloidal composites from surface-functional porous polymer microspheres. Polymer.

[34] Shim JW (2002). Zinc oxide/polymethylmethacrylate composite microspheres by *in situ* suspension polymerization and their morphological study. Colloids Surf., A..

[35] Kim YJ (2004). Synthesis and adsorption properties of gold nanoparticles within pores of surface‐functional porous polymer microspheres. J. Polym. Sci. Pol. Chem..

[36] Lee H, Dellatore SM, Miller WM, Messersmith PB (2007). Mussel-inspired surface chemistry for multifunctional coatings. Science.

[37] You I (2012). Polydopamine Microfluidic System toward a Two‐Dimensional, Gravity‐Driven Mixing Device. Angew. Chem. Int. Ed..

[38] Ryu JH (2011). Catechol-functionalized chitosan/pluronic hydrogels for tissue adhesives and hemostatic materials. Biomacromolecules.

[39] Steenken S, Neta P (1982). One-electron redox potentials of phenols. Hydroxy-and aminophenols and related compounds of biological interest. J. Phys. Chem..

[40] Potts RA (1991). Alcoholysis of nitriles in gold (III) complexes: The structure of [EtC (OEt) NH 2] + [AuCl 4]−. Polyhedron.

[41] nee Kamaldeep KS, Kaur S, Bhalla V, Kumar M, Gupta A (2014). Pentacenequinone derivatives for preparation of gold nanoparticles: facile synthesis and catalytic application. J. Mater. Chem. A.

[42] Brunauer S, Emmett PH, Teller E (1938). Adsorption of gases in multimolecular layers. J. Am. Chem. Soc..

[43] Barrett EP, Joyner LG, Halenda PP (1951). The determination of pore volume and area distributions in porous substances. I. Computations from nitrogen isotherms. J. Am. Chem. Soc..

[44] Cheng C (2012). The hydrodynamic permeability and surface property of polyethersulfone ultrafiltration membranes with mussel-inspired polydopamine coatings. J. Membr. Sci..

[45] Gao A, Liu F, Xue L (2014). Preparation and evaluation of heparin-immobilized poly (lactic acid) (PLA) membrane for hemodialysis. J. Membr. Sci..

[46] Kim I, Son HY, Yang MY, Nam YS (2015). Bioinspired design of an immobilization interface for highly stable, recyclable nanosized catalysts. ACS Appl. Mater. Interfaces.

[47] Lee Y, Park TG (2011). Facile fabrication of branched gold nanoparticles by reductive hydroxyphenol derivatives. Langmuir.

[48] Kim JS, Kim TG, Kong WH, Park TG, Nam YS (2012). Thermally controlled wettability of a nanoporous membrane grafted with catechol-tethered poly (N-isopropylacrylamide). Chem. Commun..

[49] Ho CC, Ding SJ (2014). Dopamine-induced silica–polydopamine hybrids with controllable morphology. Chem. Commun..

[50] Niedrig H, Rau EI (1998). Information depth and spatial resolution in BSE microtomography in SEM. Nucl. Instr. Meth. Phys. Res. B.

[51] Janz A, Köckritz A, Yao L, Martin A (2010). Fundamental calculations on the surface area determination of supported gold nanoparticles by alkanethiol adsorption. Langmuir.

[52] Ichimura K, Funabiki A, Aoki KI, Akiyama H (2008). Solid phase adsorption of crystal violet lactone on silica nanoparticles to probe mechanochemical surface modification. Langmuir.

[53] Joseph V (2011). SERS enhancement of gold nanospheres of defined size. J. Raman Spectrosc..

[54] Adak A, Bandyopadhyay M, Pal A (2005). Removal of crystal violet dye from wastewater by surfactant-modified alumina. Sep. Purif. Technol..

