# Supplementary material for: Bioinspired Synthesis of Mesoporous Gold-silica Hybrid Microspheres as Recyclable Colloidal SERS Substrates
Source: Sci Rep. 2017 Nov 7;7:14728. doi: 10.1038/s41598-017-15225-8 (PMC5676677; doi:10.1038/s41598-017-15225-8)
Supplement: Supplementary file 1 — Supplementary information [file 41598_2017_15225_MOESM1_ESM.pdf]

Supplementary Information

Bioinspired Synthesis of Mesoporous Gold-silica Hybrid Microspheres as Recyclable Colloidal SERS Substrates

Ho Yeon Son[1], Kyeong Rak Kim[1], Jun Bae Lee[2], Trang Huyen Le Kim[1], Jihui Jang[2], Su Ji Kim[2], Moung Seok Yoon[2], Jin Woong Kim[3,4,*], and Yoon Sung Nam[1,5,*]

[1]Department of Materials Science and Engineering, Korea Advanced Institute of Science and Technology, 291 Daehak-ro, Yuseong-gu, Daejeon, 34141, Republic of Korea

[2]Innovation Lab, Cosmax Research & Innovation Center, 662 Sampyong-dong, Bundang-gu, Seongnam, Gyeonggi-do, 13486, Republic of Korea

[3]Department of Bionano Technology, Hanyang University, 55 Hanyangdaehak-ro, Sangnok-gu, Ansan, Gyeonggi-do, 15588, Republic of Korea

[4]Department of Applied Chemistry, Hanyang University, 55 Hanyangdaehak-ro, Sangnok-gu, Ansan, Gyeonggi-do,15588, Republic of Korea

[5]KAIST Institute for the NanoCentury, Korea Advanced Institute of Science and Technology, 291 Daehak-ro, Yuseong-gu, Daejeon, 34141, Republic of Korea

* To whom correspondence should be addressed.
E-mail: yoonsung@kaist.ac.kr (Y.S.N.) and kjwoong@hanyang.ac.kr (J.W.K.) ; phone: +82-42-350-3311; fax: +82-42-350-3310

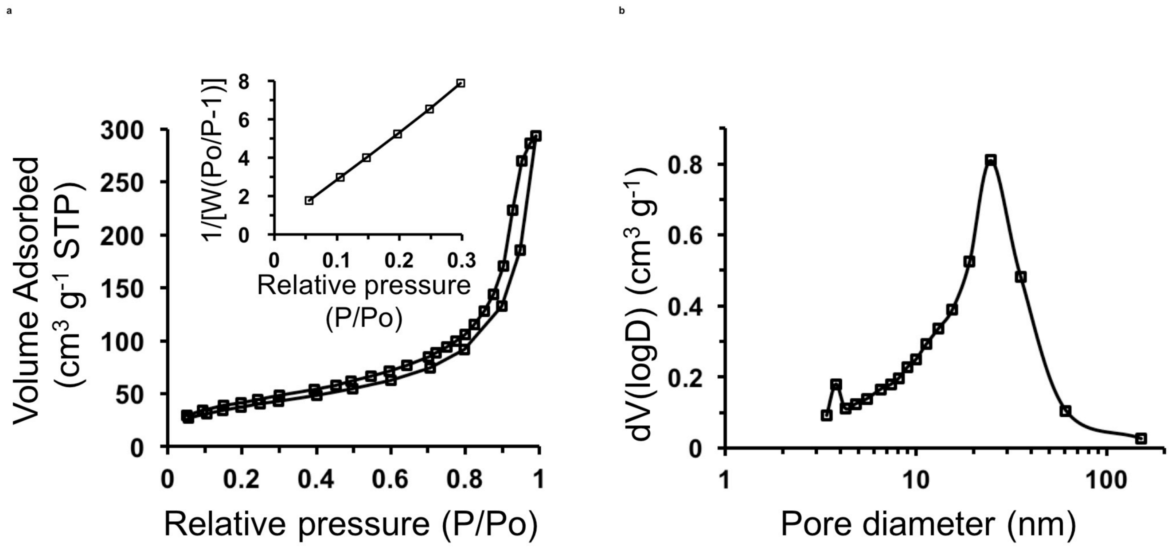

Figure S1. Isotherms of N_2 adsorption (a, inset: BET surface area plots), and pore volume distribution (b) of the synthesized mesoporous poly(EGDMA-co-AN) microspheres.

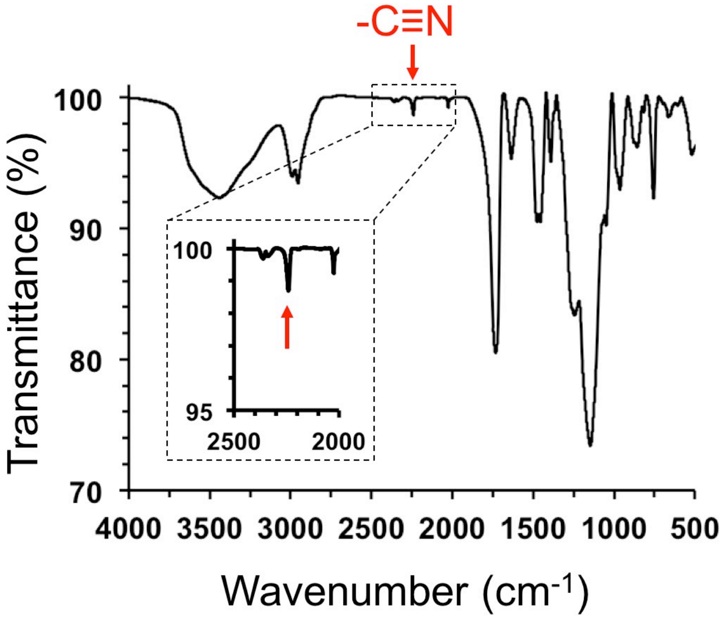

Figure S2. FT-IR spectrum of the synthesized mesoporous poly(EGDMA-co-AN) microspheres.

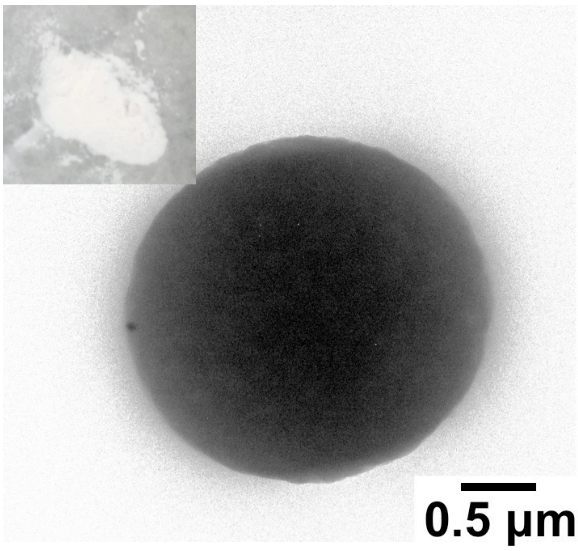

Figure S3. TEM image of gold-poly(EGDMA-co-AN) hybrid microspheres prepared by method 1 using the pristine microspheres instead of the pD-coated microspheres as a negative control. (Inset: photograph of the dried powders)

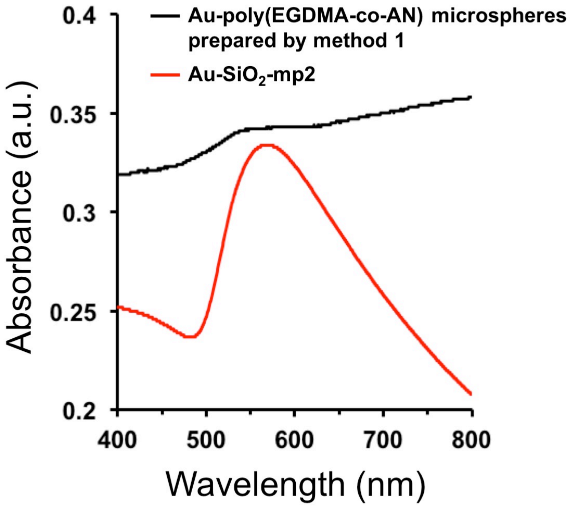

Figure S4. UV-Vis absorption spectra of the Au-poly(EGDMA-co-AN) microspheres prepared by Method 1 and the Au-SiO_2-mp2 dispersed in deionized water.

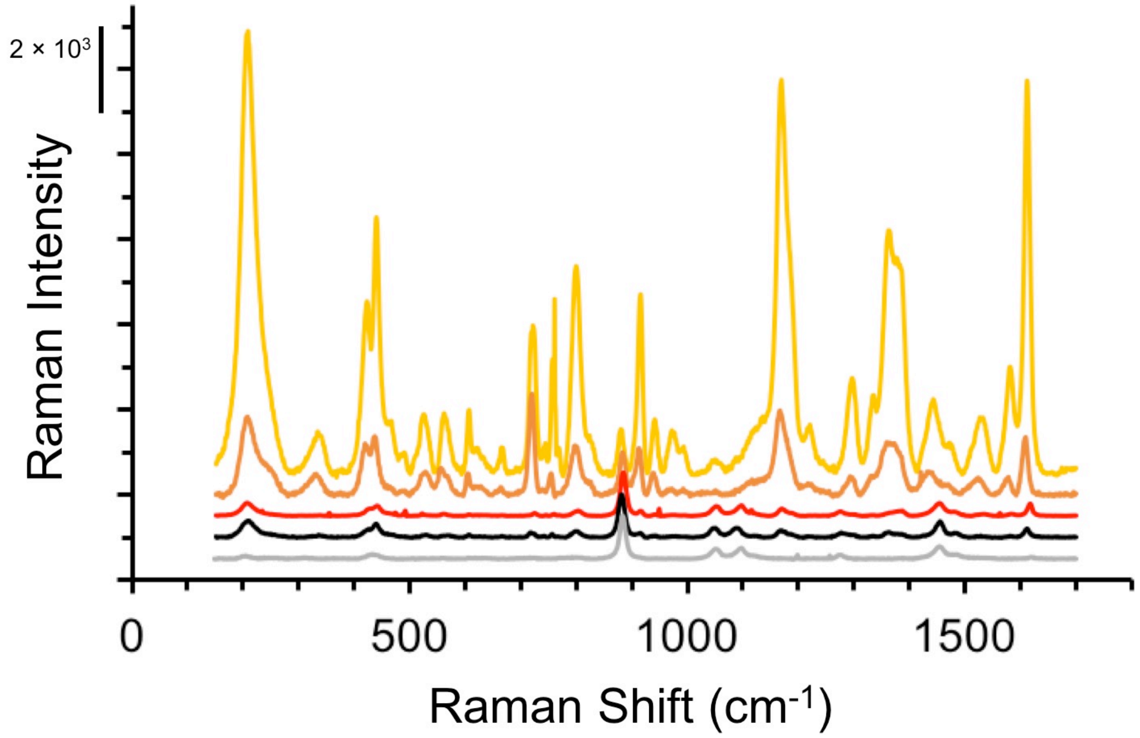

Figure S5. SERS spectra of 10^{-6} M CV with Au-SiO_2-mp2 at different gold concentrations ([Au^0] = 2 mM (yellow), 1 mM (orange), and 0.5 mM (red)) and with citrate-stabilized AuNPs ([Au^0] = 0.5 mM, black). Gray line indicates a SERS spectrum of 10^{-5} M CV solution.

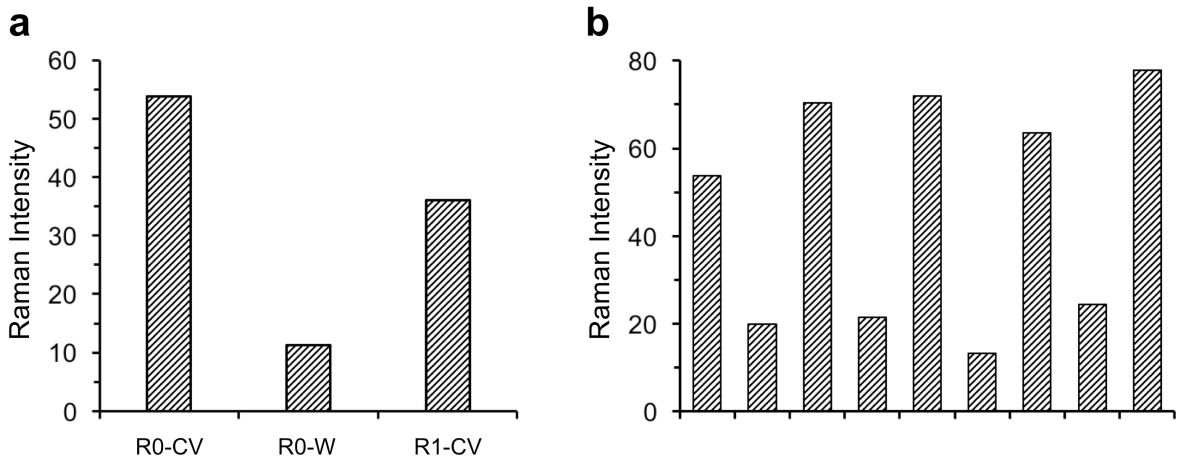

Figure S6. Characterization of reusability of Au-SiO_2-mp2 for the detection of 10^{-7} M CV. Each SERS intensities at 206 cm^{-1} using reusable Au-SiO_2-mp2 washed with ethanol containing NaOH for 2 h (a) and 10 min (b).

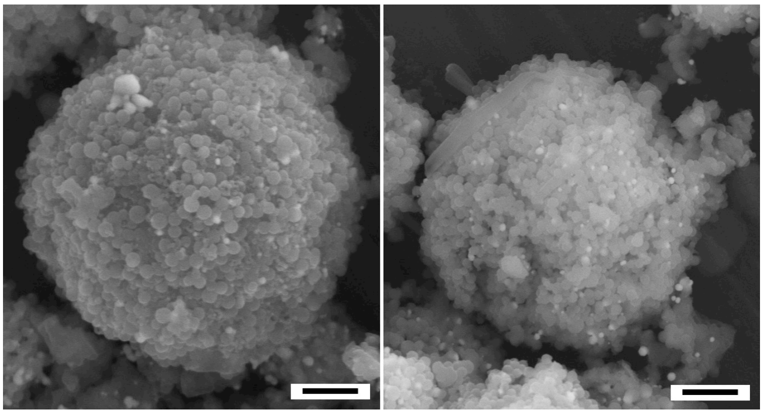

Figure S7. SEM images of the Au-SiO_2-mp2 after exposure to NaOH for 2 h.
